# The Synthesis and Properties of Liquid Crystalline Polyurethanes, Chemically Modified by Polyhedral Oligomericsilsesquioxanes

**DOI:** 10.3390/molecules24224013

**Published:** 2019-11-06

**Authors:** Artur Bukowczan, Edyta Hebda, Maciej Czajkowski, Krzysztof Pielichowski

**Affiliations:** 1 Department of Chemistry and Technology of Polymers, Cracow University of Technology, Warszawska 24, 31-155 Kraków, Poland; ehebda@chemia.pk.edu.pl (E.H.); kpielich@pk.edu.pl (K.P.); 2 Łukasiewicz Research Network—PORT Polish Center for Technology Development, Stabłowicka 147, 54-066 Wrocław, Poland; Maciej.Czajkowski@port.org.pl

**Keywords:** liquid crystalline polyurethanes, POSS, hybrids, structure, thermal properties

## Abstract

In this work, we report for the first time on the influence of polyhedral oligomericsilsesquioxanes (POSS) on the structure and properties of liquid crystalline polyurethane (LCPU). LCPU/POSS hybrids were synthesized via a two-step method. In the first step, 4,4′-methylenephenyl diisocyanate (MDI) and polytetramethylene ether glycol (PTMG) reacted with functionalized trisilanolphenyl POSS (TSP-POSS) bearing three hydroxyl groups. In the second step, the growing chain was extended with 4,4′-bis(hydroxyhexoxy)biphenyl (BHHBP). FTIR measurements confirmed the chemical bonding between the POSS and LCPU matrix and showed the influence of the silsesquioxane modification on the intensity of hydrogen bonds. The DSC and POM techniques confirmed the formation of liquid crystalline phases. The incorporation of silsesquixanes into the LC matrix leads to higher melting and isotropization temperatures along with the broadening phase transition effect. Scanning electron microscopy showed a good distribution of POSS moieties, both in the bulk and on the surface of the liquid crystalline PU matrix, whereby wide-angle X-ray diffraction (WAXD) patterns revealed halos from both the liquid crystalline and unmodified polyurethane matrix. The stress at the breaking points for LCPU/POSS hybrids containing 50% and 60% of elastic segments is greater than the stress at the breaking point of the reference material (LCPU), what is due to good dispersion of POSS in less elastic matrix. Thermal properties of the LCPU/POSS materials obtained, determined by TGA, revealed that the char residue increased with the amount of POSS for 40% of elastic segments materials.

## 1. Introduction

The synthesis and modification of thermotropic liquid crystalline polymers by applying different types of substrates and additives has been widely studied over the last decade [1]. One of the promising materials that is of particular interest is polyurethane (PU), which combines advantageous mechanical properties with good thermal stability [2]. Moreover, vast possibilities of structure modifications allow synthesizing PU with desired properties, such as biocompatible or thermally resistant materials. However, the syntheses of high-performance liquid crystalline polyurethanes (LCPUs) meet difficulties caused by the presence of hydrogen bonding of urethane groups, interactions and orientations of rigid and elastic segments, and phase separation [3,4]. The application of nanoparticles, as revealed in recent developments [5,6], could help to enhance liquid crystallinity via specific interactions on the nanoscale. Nanoparticles applied, such as carbon nanotubes and nanowires [7,8,9,10], show high synergy with the liquid crystalline phase. Recently, it has been found that a combination of polymer matrix, liquid crystalline additive, and nanoparticles may lead to novel, photoinduced actuators with unique properties. Azobenzene liquid crystalline material can be used to enhance the control of photo-mechanical bending deformations, shape recovery on heating, and other light-responsive properties. It was also shown that liquid crystalline properties can be successfully combined with graphene oxide nanoparticles, leading to novel biomimetic materials [11,12,13]. However, polyhedral oligomericsilsesquioxanes (POSS), owing to their remarkable features—combining organic and inorganic units that allow for creating chemical bonds with polymeric matrixes—may have a beneficial impact on the development of the liquid crystalline phase. It has been found that the incorporation of silsesquioxane molecules into a polyurethane leads to phase separation of the hard and soft domains in the PU matrix, which is a crucial factor in the LC phase formation [14,15,16]. For instance, Wang et al. [17] observed that doping a liquid crystalline azobenzene matrix with vinylated POSS leads to higher smectic-nematic and nematic-isotropic transition temperatures than in the neat material, and this effect is caused by the presence of a rigid cage-like silsesquixane structure. In another work, an increase was found of the vertical alignment of POSS with a liquid crystalline cyanobisphenyl host, as small amounts of nanoparticles were found [18]. Recently, other papers concerning POSS-LC interactions have been published [19,20], but to the best of our knowledge, liquid crystalline polyurethanes containing POSS not yet been described.

In our work, we used 4,4′-methylenephenyl diisocyanate (MDI) and polytetramethylene ether glycol (PTMG), and—as a chain extender—BHHBP to form side-chain polyurethanes with liquid crystalline properties. A low molar mass of the polyol (Mw = 650) was selected for better LC properties in the PU matrix [21]. The mesogenic chain extender with six methylene groups (spacers) between the aromatic core and hydroxyl functional groups was chosen for offering good LC phase formation [22]. The majority of research on LCPU is focused on the solution method with a high amount of the solvent. However, our work describes a bulk method with a solvent in the form of *N*,*N*-dimethylformamide (DMF) added only to decrease the viscosity and dissolve the chain extender. As a reactive nanofiller, trisilanolphenyl POSS (TSP-POSS) was chosen, due to its structure containing aromatic rings on the corners of the POSS cage; and it was hypothesized that such a molecule may have an influence on the formation of hydrogen bonds by causing steric hindrance. The single cage diameter of such nanoparticles is ca. 1–3 nm [22]. We investigated the influence of the elastic segment (SE) amount and the POSS addition on the formation of liquid crystallinity in the PU matrix. The crystallinities and morphologies of the resultant nanocomposites were also studied.

## 2. Results and Discussion

### 2.1. Characterization of 4,4′-Bis(hydroxyhexyloxy)bisphenyl (BHHBP)

**Figure 1:**
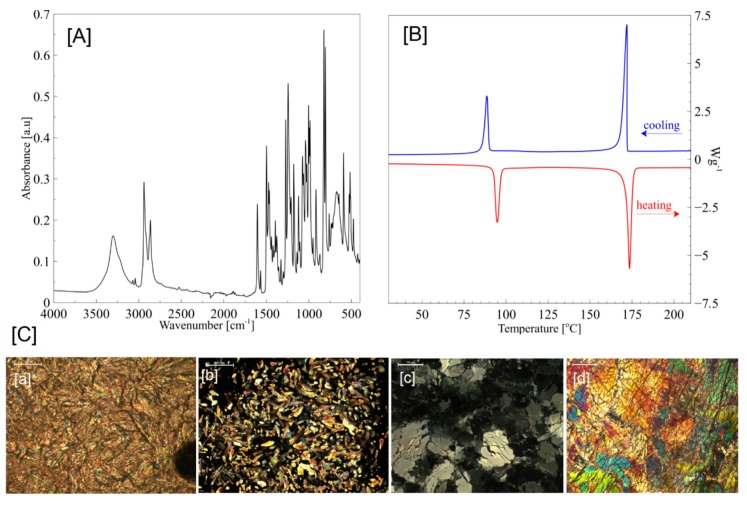
(**A**) FTIR ATR, (**B**) DSC, and (**C**) POM (a—90 °C; b—176 °C; c—90 °C cooling; d—50 °C cooling) characteristics of BHHBP mesogen; magnification 200 µm.

The chemical structure of BHHBP was confirmed by the FT-IR (ATR) spectra. The following characteristic bands were observed: 3260 cm^−1^ OH, 3029 cm^−1^ Ar–H, 2923 cm^−1^ asymmetrical CH_2_, 2805 cm^−1^ symmetrical CH_2_, 1605 cm^−1^ and 1496 cm^−1^ Ar, 1440 cm^−1^ O–CH_2_, 1270 cm^−1^ C_alif_-OH, 1240 cm^−1^ C_alif_–O–C_aromat_, 820 cm^−1^ C_aromat_–H—Figure 1A.

BHHBP creates strong birefringent liquid crystalline textures. Mosaic textures, characteristic for highly-ordered smectic phases, were observed. In the cooling segment 125–120 °C, the smectic phase (Sm2) exhibited mosaic textures. A slight change of the textures appeared in the temperature range 100–90 °C. According to the literature [23], dendric growth and similar texture are characteristic for the SmB, SmG, and SmH phases—Figure 1C.

In the heating mode, two endothermic phase transitions, characterized by enthalpy changes of 52 and 102 J/g, were observed. The first peak is associated with crystalline-to-smectic phase transition and the second one represents a smectic-to-isotropic change. Moreover, in the cooling mode, two transitions occurred with exothermic enthalpy changes of 104 and 45 J/g. The BHHBP smectic phase appears in both the heating and cooling segments, which confirms the enantiotropic properties—Figure 1B.

### 2.2. Characterisation of LCPU/POSS Nanohybrids

**Figure 2:**
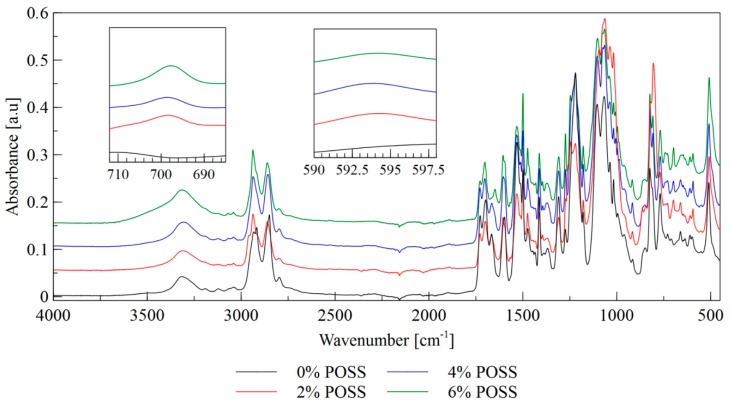
FTIR spectra of LCPU/POSS materials.

The LCPU/POSS hybrids obtained were characterized by the means of IR analysis—Figure 2. FT-IR spectral data are shown in Table 1.

The bands at 3300 cm^−1^ correspond to the stretching vibrations of hydrogen bonded NH groups. As the absorption bands at 3500 cm^−1^, associated with the stretching vibrations of the free NH groups, did not occur, it may be concluded that all the NH groups participate in the formation of hydrogen bonds. The bands at 2950 cm^−1^ and 2850 cm^−1^ correspond to the asymmetric and symmetric stretching vibrations of CH groups, respectively. The POSS load does not affect the aforementioned bands significantly. The bands at 1720–1660 cm^−1^ are due to the stretching vibrations of urethane carbonyl groups. The bands in the wavenumber range of 1150–1030 cm^−1^ are associated with the asymmetric stretching vibrations of ether linkages C–O–C that are present in the soft segments derived from the polyol component. The bands observed at 1100 cm^−1^ are correlated with the stretching vibrations of free ether groups in the soft segment, while the bands at 1040 cm^−1^ correspond to the ether groups that participate in the formation of hydrogen bonds. Those bands are overlapped with the bands associated with the stretching vibrations of the Si–O–Si groups present in the silsesquioxane cage. The stretching vibrations of Si–O bonds are also represented by the bands observed in the wavenumber range of 610–590 cm^−1^. As shown in Figure 2, the intensity of this band increases with an increase of the POSS content. The bands at approximately 700 cm^−1^ arise from the stretching vibrations of Si–C bonds that are formed between a silicon atom and methyl group in the POSS cage. The highest intensity of this band is observable for the material with the highest POSS content. The bands observed in the spectral range 1260–1230 cm^−1^ correspond to C=N stretching vibrations. Those bands overlap with the bands associated with the stretching vibrations of CH_2_OH groups (derived from polyol component and chain extender) that are usually observed at 1250 cm^−1^. The bands at a wavenumber of 2250 cm^−1^, originating from the stretching vibrations of isocyanate groups, are absent, which confirms that the isocyanate substrate completely reacted.

**Figure 3:**
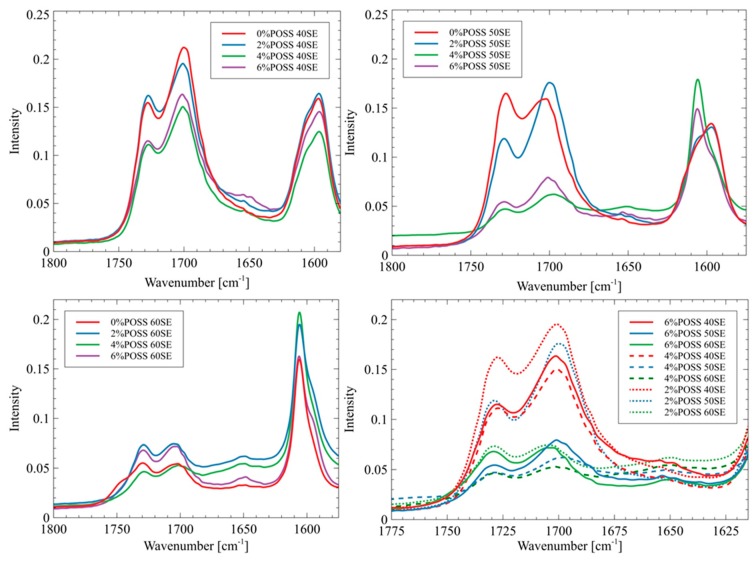
FTIR spectrum of carbonyl area in liquid crystalline polyurethane/POSS hybrids.

The absorption bands at 1700 cm^−1^ correspond to the stretching vibrations of the ordered hydrogen bonded carbonyl groups present in the urethane linkages, while the bands at 1725 cm^−1^ are associated with the stretching vibrations of the free carbonyl groups [24,25]. The presence of the free carbonyl groups indicates that the microphase separation in the materials was incomplete. The band observed at 1650 cm^−1^ corresponds to the vibration of the disordered carbonyl groups that are hydrogen bonded in the soft phase, which also indicates incomplete phase separation (Figure 3).

The intensity of the bands at 1650 cm^−1^ is stronger for the materials that contain POSS than for the reference materials. This may indicate that the presence of POSS disrupted the microphase separations, and thus the phase mixing process was observed. Such an influence of POSS moieties on the polyurethane phase’s behavior has been reported by Raftopoulos et al. The restriction of the microphase separation by POSS was explained by the silsesquioxane presence hindering the formation of the hard segments’ domains [26]. The non-linear influence of the POSS content on the morphology of the polyurethane elastomers, which has been found by Raftopoulos et al., was also observed. Lewicki et al. [27] have shown that the introduction of the POSS molecules, which are nonpolar and sterically bulky, disrupts the forces driving the microphase separation. As a result, the crystallinity degree of the matrix decreases with an increase of the POSS content. The origin of this effect of silsequioxane on the microphase morphology in relation to the impact on the chemical potential differences of the two types of segments and on the enthalpic driving force of the crystallization of the hard domains is that POSS moieties act as a counter-factor to both of the forces governing the phase separation. Thus, the presence of POSS results in lower phase separation and more amorphous state of the materials [28].

**Figure 4:**
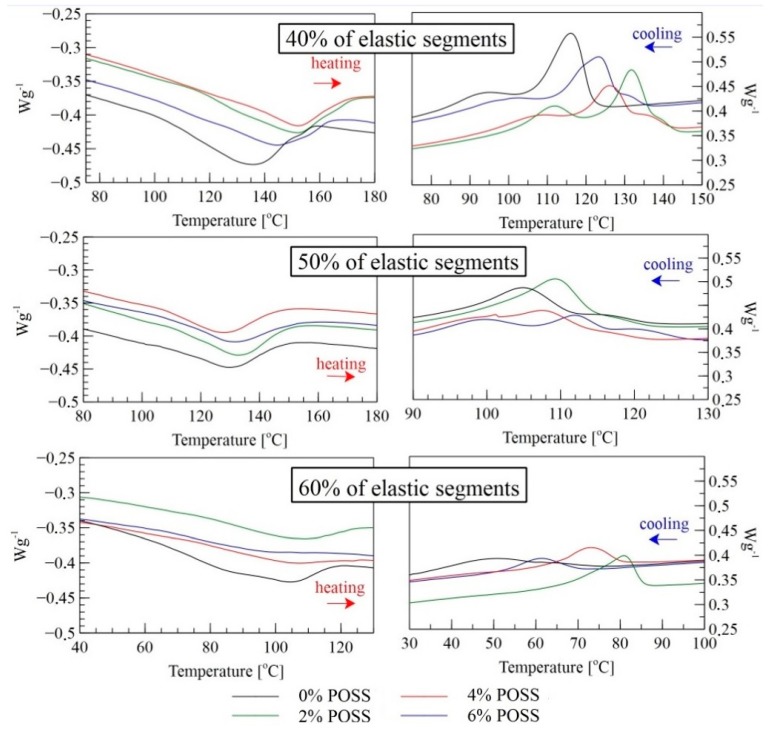
DSC profiles of LCPU/POSS materials: second heating and cooling cycle.

The thermal transition temperatures of the LCPU and LCPU/POSS nanocomposites were studied by DSC—Figure 4.

In the heating mode, all the samples undergo a melting process at different temperature ranges depending on the soft/hard segment ratio and the POSS load. The PU with 40% of elastic segments showed the highest range of thermal transition due to the highest amount of the mesogenic chain extender (Table 2). (T_i–lc_—temperature of transition from isotropic to liquid crystalline phase; T_lc–k_—temperature of transition from liquid crystalline to crystalline).

The addition of POSS shifts that thermal effect towards higher temperatures and causes a broader temperature range of melting. In the cooling mode, for all the modified matrices, the temperature transition from isotropic to liquid crystalline was distinct and shifted towards higher temperatures. Furthermore, larger amounts of POSS lead to a separation of two peaks into three, with a lower enthalpy-change effect. This phenomenon can be explained by the existence of two mesophases of different thermodynamic stability in LCPU. The melting behavior of the model compounds suggests that this effect might be related to the existence of MDI and BHHBP isomeric structures. Nevertheless, it was not possible to distinguish such textures by polarized optical microscopy. The highest thermal effects for 40% and 60% elastic segments in the cooling mode were observed for a 2% load of POSS, whereas for 50% of elastic segments, the best effects were observed for 6% of the POSS load. This effect might be cause of higher crystallinity (Table 5) for those samples where the effect of liquid crystalline transition can overlap the effect of crystallization. The influence of POSS on liquid crystalline properties highly depends on hard/soft segments ratio. For 40% and 60% elastic segments, the most appropriate load of POSS is 2 wt%. For equal amounts of segments, the highest value of POSS gives better liquid crystalline properties. All the effects shown in the DSC thermograms can be combined with the FTIR results of the hydrogen bonds formation after the incorporation of POSS to the PU structure. The lower intensity of hydrogen bonds with carbonyl groups may have an impact on liquid crystalline phase’s formation, leading to a higher range of phase transitions.

**Figure 5:**
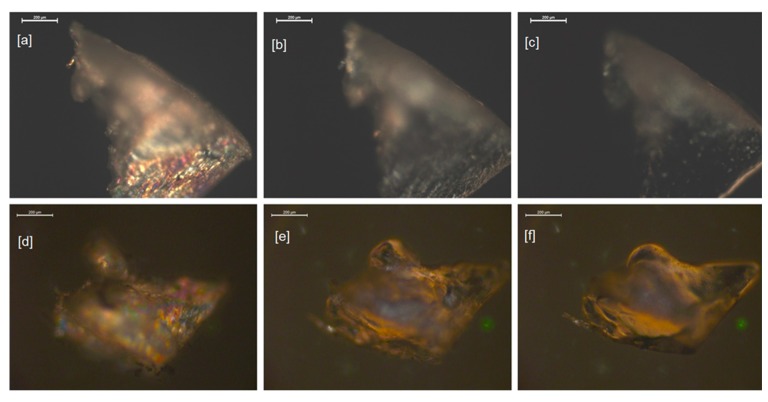
Polarized optical microscope photographs of the sample of the elastomers: 0% POSS 40SE at 40 °C (**a**), at isotropization temperature 155 °C (**b**), and near the flow temperature of 167 °C (**c**); 2% POSS 40SE at 40 °C (**d**), at isotropization temperature 160 °C (**e**), and near the flow temperature of 170 °C (**f**); magnification 200 µm.

A polarized optical microscope was used to determine the extent of birefringence of the liquid crystalline nanocomposites with respect to temperature. It was observed that the liquid crystalline phase was dispersed uniformly as a polydomain texture and its fraction was decreasing continuously with an increase of temperature—Figure 5.

At the first stage, the samples were heated up to 190 °C, above the isotropization and flow temperatures of the elastomers under investigation. The shapes of the materials changed and the recovery of the optical anisotropy of the samples to a large extent ceased after cooling the samples to room temperature. In the second stage, the elastomers were subjected to lower temperatures and it was observed that by avoiding the flow temperatures of the samples, the reversibility of the optical anisotropy was enhanced. The isotropization temperatures determined based on the microscopic measurements were comparable to the end temperatures of the peaks in the DSC heating cycle thermograms. The flow temperatures (Tflow) were determined as temperatures at which the initial stages of the liquefaction of the samples occurred, indicated by substantial smoothing of the surface and the edges of the samples, followed by changes of the shape identified on the microphotographs. The results are summarized in Table 3. The microscopic determinations of the transition temperatures were affected by uncertainty of the following origins: gradient of the temperature along the height of the sample placed in the microscopic heating stage and subjective evaluation of the transition points during the analysis of the microphotographs.

The temperatures at which change of the shape and liquefaction were observed are shown in Table 3.

**Figure 6:**
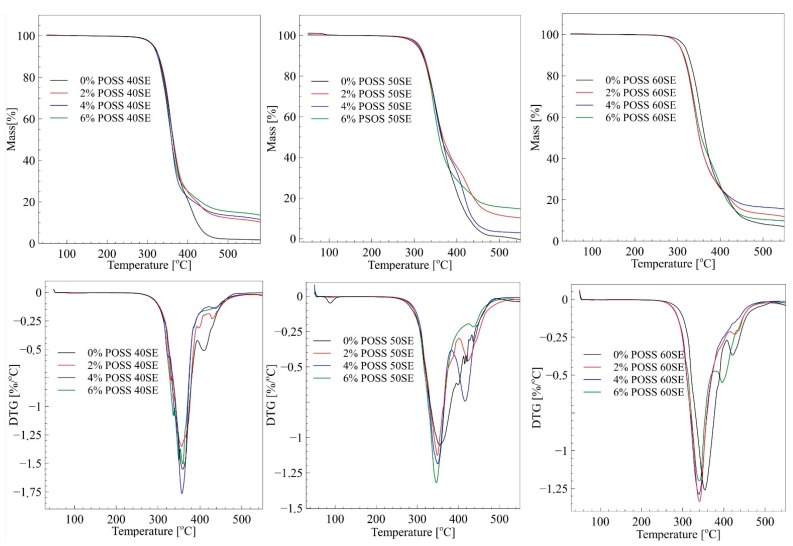
TGA and DTGA curves of LCPU/POSS materials.

TGA and DTGA results are presented in Figure 6.

Figure 6 shows the results of TG measurements for pure PU and PU/POSS composites with different POSS concentrations. For 40% elastic segments, samples’ incorporation of POSS moieties does not change thermal stability significantly, although the highest temperature in the first step of thermal degradation was observed for the highest load of POSS—Table 4. Interestingly, the improvement of thermal properties was observed for composites with 50% elastic segment content. The initial temperature of thermal degradation after doping with POSS increased by 7–9 °C. On the contrary, the 60% elastic segments matrix, after a load of POSS particles, exhibited lower thermal stability than the neat material. It can be concluded that LCPU elastomers with equal amounts of elastic and rigid segments can to be successfully thermally stabilized by POSS molecules. DTGA curves show a two-step degradation mechanism typical for segmented polyurethanes. It was observed that POSS did not significantly alter the degradation mechanism. According to the literature, decomposition of segmented polyurethane is characterized by complex mechanism, including depolymerization, cleavage of urethane bonds, and the production of volatiles such as amines, olefins, and carbon dioxide [28]. Generally, the first peak at the temperature around 350 °C corresponds to decomposition of rigid segments in LCPU matrix. The second stage of thermal degradation, represented by a smaller peak, is associated with the decomposition of oligodiol chains originating from elastic segments. The residue at the end of TG measurements increases with amount of POSS for 40% of elastic segments materials. The same trend can be observed for 50% and 60% of elastic segments; however, 4% POSS 50SE and 6% POSS 60SE shows a significant decrease of residue after the thermal decomposition. Noteworthy, is that a high amount of residue can indicate a material’s reduced flammability, as such a residue can form a thin layer on the sample surface, hindering oxidation of the material during burning.

**Figure 7:**
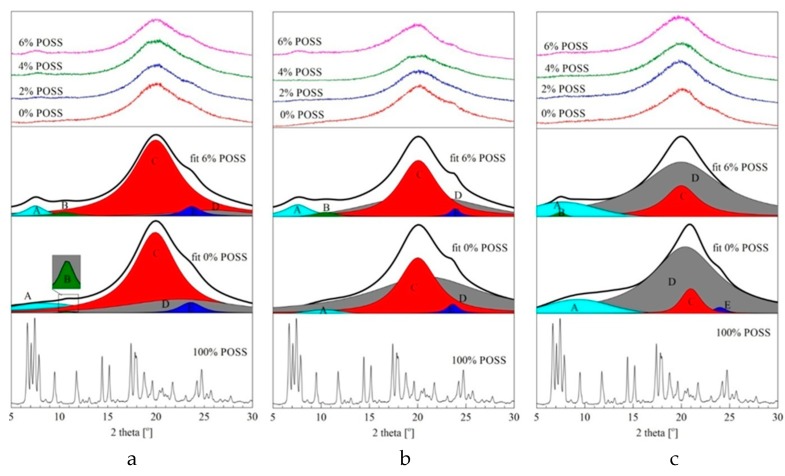
WAXD patterns for LCPU/POSS nanohybrids and neat TSP-POSS: (**a**) 40% SE, (**b**) 50% SE, and (**c**) 60% SE.

WAXD measurements were conducted after annealing the materials at 130 °C for 24 h in order to achieve the liquid crystalline phase transition temperature (Figure 7).

The detailed patterns were obtained using the curve-fitting technique based on the Gaussian and Lorentzian assumptions. According to the literature [29], there are four maxima characteristic for the supramolecular polyurethane structure: a 4–11° prepeak of an amorphous halo corresponding to the dispersion of rigid segments in the soft phase [A], a 12–30° sharp peak related to the distances between rigid segments [C], and a broad amorphous halo associated with distances between flexible segments in the elastic phase. The incorporation of highly crystalline BHHBP as a chain extender caused the appearance of two additional peaks: in the range of 8–10°—a small flat peak corresponding to rigid MDI–BHHBP structures dispersed in the soft phase [B], and a sharp peak 24–26° related to the distance between MDI–BHHBP chains in the rigid phase [E]. The intensity of the amorphous halo increases with a growing amount of elastic segments, and is accompanied by a smaller decrease of the peak related to the hard domain distances. The crystalline peaks from the POSS agglomerates are not visible in the WAXD patterns, and that was confirmed by the SEM microphotographs (Figure 9). The higher intensity of the pre-peak [A] for the 6% POSS composites is most likely an effect of a higher value of the rigid domain blocks built by MDI–POSS–MDI repetitive units dispersed in the soft phase. Moreover, the main amorphous halo shifts slightly towards the lower value of two theta angles due to crosslinking behavior of the TSP–POSS moieties and a decrease of the interfacial distances. The degree of crystallinity based on the curve-fitting technique was calculated, and crystallinity increased with an increasing amount of the hard segments capable of forming ordered crystalline phases, as seen in Table 5. Interestingly, the largest value of crystallinity was exhibited by the 2% load of POSS in 40% elastic segment matrix. It can be assumed that TSP–POSS in low amounts facilitates the nucleation and formation of higher-ordered crystalline structures.

**Figure 8:**
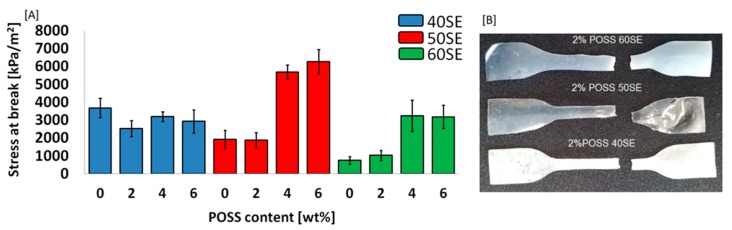
Stress at break measurement (**A**), and a digital image (**B**) for LCPU/POSS materials.

With the objective to examine the influence of POSS molecules on the mechanical properties of a liquid crystalline matrix, stress at break was measured—Figure 8A. Digital images of LCPU films are presented in Figure 8B.

**Figure 9:**
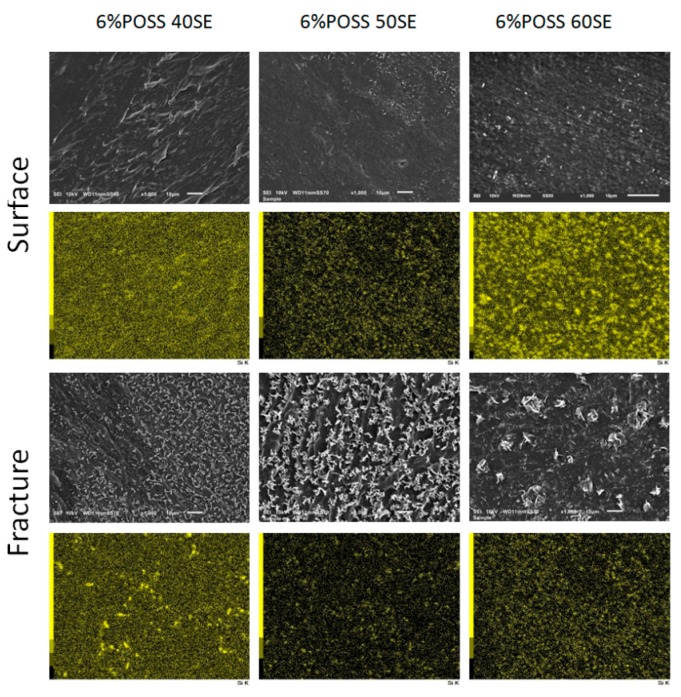
SEM images of LCPU/POSS nanocomposites on the surface and in the bulk of sample after fracturing (magnification 1000×, scale on the images 10 μm) and EDS mapping where SEI images are linked with a silicon map.

The reinforcement properties of TSP-POSS were observed for the samples with 50% and 60% elastic segments. Such an effect is a result of good dispersion of POSS in a less elastic matrix. According to SEM microphotographs (Figure 9), the distribution of nanoparticles was homogenous. In contrast, the samples containing 40% elastic segments after the incorporation of POSS particles exhibited lower stress at break. It may be result of a high level of crystallinity, in particular for the 2 wt% POSS load, which makes the material durable but at the same time more brittle.

In order to investigate the POSS distribution in the LCPU matrix, SEM microphotographs were taken on the surface and in the bulk of the materials after fracturing—Figure 9.

It was observed that POSS nanoparticles are well dispersed on both the surfaces and in the interiors of the samples. Some POSS crystallites were found for the material containing 40% elastic segments, which may be a result of higher rigidity disturbing the homogenous distribution of nanoparticles in the polymer matrix.

## 3. Materials and Methods

4,4′-dihydroxybiphenyl (BHBP) and 6-chloro-1-hexanol (97%) were purchased in Alfa Aesar and used as received. Anhydrous ethanol, sodium hydroxide, anhydrous *N*,*N*-dimethylformamide, 4,4′-methylenephenyldiisocyanate, and 1-butanol were acquired from Sigma Aldrich. MDI was stored in an inert gas atmosphere in a freezer. TSP-POSS was produced by Hybrid Plastics and stored at a low temperature. PTMG with a molecular mass of 650 and 1,4-butandiol were dried under vacuum for 24 h at 80 °C.

### 3.1. Synthesis of Mesogenic Diol

4,4′-bis(hydroxyhexoxy)biphenyl (BHHBP) was synthesized based on the procedure described by Lee et al. [30] with some modifications. In total, 250 mL of anhydrous ethanol was placed into a triple-necked flask equipped with a dropping funnel, thermometer, and air condenser and heated to 70 °C. It was discovered that the use of anhydrous alcohol increased, significantly, the yield of the reactions. In the next step, BHBP (19 g) and NaOH (16 g) were added while being stirred vigorously under reflux. An excess of 6-chloro-1-hexanol (60 g) was then added dropwise for 2 h. The reaction was carried out for 24 h and then the product was precipitated into cold, demineralized water. The white powder obtained in the experiment was purified by recrystallization, first from an ethanol/DMF mixture, and then from 1-butanol. The yield of the process was 70–76% and the melting point of the products was 70.1–70.6 °C.

### 3.2. Synthesis of the Liquid Crystalline PU/POSS Hybrids

**Figure 10:**
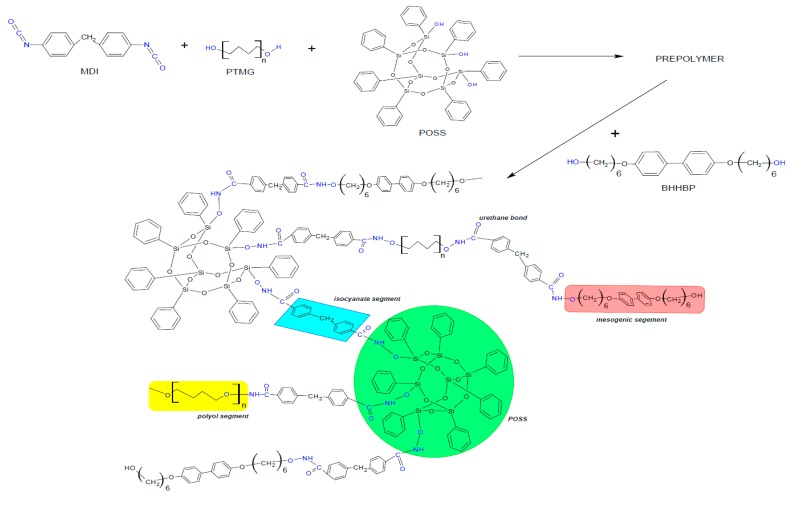
Scheme of liquid crystalline polyurethane (LCPU)/POSS hybrid synthesis route.

Three different types of LCPUR matrices were prepared via a two-step prepolymer method, containing 40%, 50%, or 60% elastic segments. Each PU system was modified with 2, 4, and 6 wt% of TSP-POSS. The reaction path was such that MDI diisocyanate was first melted at 60 °C and then 10 mL of anhydrous DMF was added. In the next step, the polyol and POSS were added to the solution, which was then stirred vigorously, and heated up to 80 °C—Figure 10. The reaction progress was monitored by FTIR by following the changes in the peak assigned to isocyanate groups at 2200 cm^−1^. The reaction was terminated by adding a mesogenic chain extender (1,4-butandiol for neat material) dissolved in anhydrous DMF at 80 °C. All of the syntheses lasted for ca. 75 min. Finally, the reaction mixture was poured into a steel mould and dried for 24 h at 120 °C.

### 3.3. Infrared Spectroscopy with Fourier Transformations (FTIR)

Infrared, spectroscopic Fourier transform measurements were performed on a Nicolet iS5 spectrometer (Thermo Fisher Scientific, Waltham, MA, USA) equipped with a diamond crystal attenuated total reflectance unit (ATR). Spectra were measured with a resolution of 8 cm^−1^ from 4000 to 400 cm^−1^, with an average of 16 scans.

### 3.4. Differential Scanning Calorimetry (DSC)

For DSC measurements, Mettler–Toledo DSC (Mettler-Toledo, Columbus, OH, USA) was applied. The DSC measurements were performed under a heating/cooling rate of 10 K/min, with two heating and two cooling segments in the temperature range 20–220 °C. All the measurement were done under a nitrogen atmosphere using an intracooler as a cooling system.

### 3.5. Polarized Optical Microscopy (POM)

POM observations were performed using a polarized microscope DM2700P (Leica, Wetzlar, Germany) equipped with a heating stage (Linkam, LTS420, Waterfiled, UK). Samples of submillimeter dimensions were cut and placed on a microscopic cover glass, and heated at a heating range of 10 K/min.

### 3.6. Wide-Angle X-Ray Diffraction (WAXD)

A Bruker D Phaser diffractometer (Bruker, Billerica, MA, USA) was used for WAXD investigations in reflection mode. A standard CuKα anode with the wavelength λ = 1.54184 Å was applied.

### 3.7. Thermogravimetric Analysis (TGA)

Measurements were performed using a TA Instruments TGA 550 Discovery (TA Instruments, New Castle, DA, USA), operating in dynamic mode at a heating rate of 10 K/min from 40 to 600 °C in an atmosphere of nitrogen. The sample mass was ca. 3.5 mg, measured in open Pt crucibles.

### 3.8. Mechanical Properties

Stress at break tests were performed based on the ISO 527-2 type 5A and ISO 37 type 2 standards. Samples were prepared in a form of thin films in a shape of “paddles.” Measurements were conducted using a Brookfield CT3 texture analyzer (AMETEK Brookfield, Middleborough, MA, USA).

### 3.9. Scanning Electron Microscopy (SEM)

Scanning electron microscopy (SEM) micrographs were taken using a JEOL InTouchScope JSM-6010LV (JEOL Ltd., Tokyo, Japan) microscope with energy-dispersive X-ray analysis (EDS) capabilities, operated at 10 kV accelerating voltage.

## 4. Conclusions

New organic—inorganic hybrid materials based on liquid crystalline polyurethanes, modified by polyhedral oligomeric silsequioxanes, were synthesized using BHHBP, which exhibits smectic liquid crystalline phases as a chain extender. The effect of POSS on liquid crystalline properties was investigated by the DSC technique and it was found that incorporation of rigid silsesquioxanes moieties with aromatic rings results in a higher phase transition temperature and a broader range of LC phenomena. The best mesomorphic properties were displayed by polyurethane with 40% elastic segments, which could lead to conclusions that this ratio of soft and rigid segments–liquid crystalline phase formation is favored. Higher POSS loads cause a separation of two DSC peaks into three, with a lower enthalpy change effect that can be explained by the existence of two mesophases of different thermodynamic stabilities in the LCPU. FTIR measurements in the absorption range of carbonyl groups showed lower intensities for the peaks responsible for hydrogen bonds, which might be a result of incomplete phase separation due to steric hindrance of the aromatic POSS structure. Incorporation of TSP–POSS does not lead to the formation of agglomerates, which was proven by scanning electron microscopy and WAXD measurements. X-ray diffraction patterns showed a shift of the main amorphous halo to lower theta angles as a result of the crosslinking properties of TSP–POSS. The nanoadditive in low amounts facilitates the nucleation and formation of higher ordered crystalline structures, as evidenced by the value of the degree of crystallinity of PU (40% elastic segments) containing 2% of silsesquioxane. Stress at break measurements revealed that the reinforcement effects of TSP–POSS were observable for polyurethane materials containing 50% and 60% elastic segments, in which a fine distribution of nanoparticles was observed by the SEM technique. On the other hand, incorporation of POSS moieties in PU matrix with 40% elastic segments lead to a lower stress at break due to an increase of the degree of crystallinity. Thermogravimetric analysis results show that the char residue increases with the amount of POSS for 40% elastic segments materials; this observation can indicate a material’s reduced flammability through the formation of a protective layer on the sample’s surface, which limits oxidation processes during burning.

## Figures and Tables

**Table 1:** FT-IR spectral data.

Frequency Range [cm^−1^]	Functional Groups	Vibration Mode
3300	N-H	stretching vibrations of hydrogen bonded NH groups
2950	C-H	asymmetric stretching vibrations
2850	C-H	symmetric stretching vibrations
1720–1660	C=O	stretching vibrations of urethane carbonyl groups
1725	C=O	stretching vibrations of the free carbonyl groups
1700	C=O	stretching vibrations of the ordered hydrogen bonded carbonyl groups present in the urethane linkages
1650	C=O	stretching vibration of the disordered carbonyl groups that are hydrogen bonded in the soft phase
1260–1230	C=N	stretching vibrations
1250	CH_2_OH	stretching vibrations
1150–1030	C-O-C Si-O-Si	asymmetric stretching vibration of ether groups in soft segments stretching vibrations of groups in POSS cage
1100	C-O-C	stretching vibrations of free ether groups in the soft segments
1040	C-O-C	stretching vibrations of hydrogen bonded ether groups
700	Si-C	stretching vibrations
610–590	Si-O	stretching vibrations

**Table 2:** Thermal effects during heating and cooling of LCPU/POSS nanohybrid material.

Sample	Heating		Cooling	
	T_m_ [°C]	T_i_ [°C]	T_i–lc_ [°C]	T_lc–k_ [°C]
0%POSS 40SE	105	150	125	72
2%POSS 40SE	120	170	143	109
4%POSS 40SE	125	170	141	96
6%POSS 40SE	115	160	136	92
0%POSS 50SE	110	145	122	94
2%POSS 50SE	109	153	124	96
4%POSS 50SE	110	135	122	90
6%POSS 50SE	115	147	128	90
0%POSS 60SE	60	115	66	33
2%POSS 60SE	90	120	85	64
4%POSS 60SE	85	122	80	65
6%POSS 60SE	82	98	69	53

T_i–lc_—temperature of transition from isotropic to liquid crystalline phase, T_lc–k_—temperature of transition from liquid crystalline to crystalline.

**Table 3:** Determination of isotropization temperatures of LCP/POSS hybrids.

%POSS	40SE	50SE	60SE	40SE	50SE	60SE
	TI [°C]	TI [°C]	TI [°C]	Tflow [°C]	Tflow [°C]	Tflow [°C]
0%	155 ± 10	140 ± 10	105 ± 10	165	152	120
2%	160 ± 10	140 ± 10	120 ± 10	170	155	125
4%	165 ± 10	145 ± 10	110 ± 10	177	160	145
6%	175 ± 10	155 ± 10	120 ± 10	180	170	Wide range 100–150

**Table 4:** TGA and DTGA data of LCPU/POSS materials.

Sample	T_1%_ [°C]	T_3%_ [°C]	T_5%_ [°C]	T_10%_ [°C]	T_50%_ [°C]	DTG max [°C]		Residue at 500 °C [%]
						1	2	
0% POSS 40SE	282.3	305.5	315.5	328.5	363.7	359.3	409.8	2.13
2% POSS 40SE	282.1	306.4	316.7	328.0	364.4	355.9	430.6	12.25
4% POSS 40SE	280.5	305.4	314.9	325.8	359.8	357.3	436.6	13.49
6% POSS 40SE	284.8	306.9	315.8	325.8	359.8	357.4	405.7	15.46
0% POSS 50SE	270.4	295.3	306.4	319.9	364.9	355.5	-	1.10
2% POSS 50SE	277.9	300.1	309.2	321.9	367.2	349.0	425.1	11.63
4% POSS 50SE	279.5	303.0	312.1	323.0	363.7	349.5	416.2	3.41
6% POSS 50SE	277.7	300.4	309.7	320.6	358.2	346.2	436.2	15.66
0% POSS 60SE	278.3	304.5	314.2	325.1	363.9	353.8	421.0	8.34
2% POSS 60SE	274.7	294.3	302.9	313.5	351.0	341.1	426.6	13.25
4% POSS 60SE	273.7	294.4	303.2	314.0	351.3	339.2	-	16.48
6% POSS 60SE	273.4	293.9	302.9	314.9	356.3	340.7	433.9	10.54

**Table 5:** Degree of crystallinity based on curve-fitting technique.

Sample	χ	Sample	χ	Sample	χ
	[%]		[%]		[%]
0%POSS 40SE	69	0%POSS 50SE	34	0%POSS 60SE	7
2%POSS 40SE	77	2%POSS 50SE	32	2%POSS 60SE	10
4%POSS 40SE	69	4%POSS 50SE	32	4%POSS 60SE	12
6%POSS 40SE	68	6%POSS 50SE	34	6%POSS 60SE	12
